# *Treponema pallidum* induces pro-inflammatory cytokine secretion in macrophages and macrophage-endothelial co-cultures

**DOI:** 10.3389/fcimb.2025.1681813

**Published:** 2025-10-17

**Authors:** Sean Waugh, Akash Ranasinghe, Lisa A. Reynolds, Caroline E. Cameron

**Affiliations:** ^1^ Department of Biochemistry and Microbiology, University of Victoria, Victoria, BC, Canada; ^2^ Department of Medicine, Division of Allergy & Infectious Diseases, University of Washington, Seattle, WA, United States

**Keywords:** syphilis, immunology, pathogenesis, macrophages, endothelial cells, *Treponema pallidum*

## Abstract

Syphilis, caused by the extracellular bacterium *Treponema pallidum* ssp. *pallidum*, is a multi-stage and systemic infection that is lifelong in the absence of treatment. Two host cell types that frequently encounter *T. pallidum* during infection are endothelial cells and macrophages; treponemes disseminate through the vasculature and cross the blood–brain and placental barriers by traversing endothelial cell barriers, and macrophages are known to be critical for clearance of *T. pallidum.* Despite the importance of macrophages in treponemal clearance and endothelial cells in treponemal dissemination, a comprehensive understanding of the cytokines secreted by *T. pallidum*-exposed macrophages in the presence and absence of endothelial cells has not yet been achieved. To address this knowledge gap, we conducted time-course cytokine secretion profiling of macrophage-differentiated THP-1 cells alone and in co-culture with human brain microvascular endothelial cells. These experiments revealed reduced IL-8 secretion and increased secretion of RANTES, soluble ICAM-1, IL-1β, MCP-1, GM-CSF, TNF, and IL-6 in *T. pallidum*-exposed macrophage monocultures and macrophage-endothelial cell co-cultures compared to the same culture conditions in the absence of *T. pallidum*. These investigations enhance our understanding of macrophage-mediated, *T. pallidum*-focused innate immune responses occurring at endothelial sites. Further, this study provides insight into pro-inflammatory mechanisms elicited after exposure to this pathogen that may contribute to endothelial junction disruption, *T. pallidum* dissemination, and syphilis symptoms.

## 1 Introduction

Infectious syphilis, caused by the sexually transmitted bacterium *Treponema pallidum* subsp. *pallidum*, is a multi-stage disease with a global prevalence of 49.7 million cases (Chen et al., 2023) and an annual incidence of 8 million cases (World Health Organization, 2024). Global congenital syphilis estimates are 661,000 cases per year, with an estimated 355,000 adverse birth outcomes (Korenromp et al., 2019). With syphilis cases reaching a 20-year high (Spiteri et al., 2019; Aho et al., 2022; Chen et al., 2023; National Overview of STDs, 2021, 2023), an enhanced understanding of *T. pallidum* pathogenesis, and the resulting host response to infection, is needed to inform syphilis vaccine development.

Syphilis presents with a diverse array of symptoms that occur during distinct clinical stages of disease. These include the development of a characteristic chancre at the initial site of infection (primary stage), followed by a disseminated rash and general malaise (secondary stage). The infection then enters an asymptomatic latent phase, which may persist for an individual’s lifetime. Approximately one-third of individuals develop symptoms associated with the tertiary stage of disease, which can include severe central nervous system or cardiovascular complications (LaFond and Lukehart, 2006). The symptoms of syphilis are thought to arise from the host’s immune response to the presence of *T. pallidum*, highlighting the dichotomy of a beneficial inflammatory immune response which aids in *T. pallidum* clearance and a detrimental inflammatory response that contributes to bacterial dissemination, tissue damage, and disease (LaFond and Lukehart, 2006; Carlson et al., 2011).

Previous investigations have shown that *T. pallidum* clearance from local sites of infection is accomplished by a Th (T helper cell) 1-mediated delayed-type hypersensitivity (DTH) immune response (Carlson et al., 2011), mediated by interferon (IFN)-γ-activated macrophages and the process of opsonophagocytosis (Lukehart and Miller, 1978; Baker-Zander and Lukehart, 1992; Hawley et al., 2017). Although this response results in local clearance of the majority of *T. pallidum*, a subpopulation escapes immune clearance and disseminates via the bloodstream to distal tissue sites (Lukehart et al., 1992; Leader et al., 2003; Giacani et al., 2010; Reid et al., 2014). These observations emphasize the central role of macrophages in the immune response to *T. pallidum*.

Endothelial cells, positioned at the inner lining of the bloodstream, are among the first host cell types engaged by invading pathogens and are important mediators of innate immune activation. Endothelial cells contribute to immunity by secreting cytokines to recruit immune cells to sites of infection, and express cell adhesion molecules that enable leukocyte binding and subsequent leukocyte extravasation (Lemichez et al., 2010; Amersfoort et al., 2022). *Treponema pallidum* disseminates systemically via the bloodstream, traversing endothelial cell-cell junctions via both paracellular and transcellular migration (Wu et al., 2017; Lithgow et al., 2021). Adhesion of *T. pallidum* to the endothelium, an interaction critical for treponemal extravasation, is mediated by binding of *T. pallidum* vascular adhesins to extracellular matrix (ECM) components and endothelial cell receptors (Cameron, 2003; Cameron et al., 2004; Brinkman et al., 2008; Lithgow et al., 2020). Endothelial cells are activated by *T. pallidum* exposure, resulting in increased expression of cell adhesion molecules (Riley et al., 1992). Additionally, endothelial cells exposed to *T. pallidum* display altered expression of proteins involved in endothelial ECM organization/composition and cell death signaling. Further dysregulation includes increased secretion of pro-inflammatory junction-disrupting cytokines and growth factors such as vascular endothelial growth factor (VEGF), interleukin (IL)-6, and IL-8, as well as decreased secretion of monocyte chemoattractant protein 1 (MCP-1) (Waugh et al., 2023). Endothelial immune and ECM signaling responses have been shown to have downstream effects on monocyte and macrophage activity (Amersfoort et al., 2022). Similarly, macrophages can have reciprocal influence on endothelial function, including remodeling the endothelial ECM, promoting angiogenesis, and inducing endothelial cell death through the secretion of growth factors, cytokines, ECM components, and proteolytic enzymes (Bergers et al., 2000; Baer et al., 2013; Tomlin and Piccinini, 2018).

Despite evidence demonstrating the importance of both macrophages and endothelial cells during *T. pallidum* infection, the molecular outcomes of *T. pallidum* interaction with these cell types are incompletely understood (Lukehart and Miller, 1978; Baker-Zander and Lukehart, 1992; Hawley et al., 2017; Wu et al., 2017; Lithgow et al., 2021). In particular, the way *T. pallidum* affects macrophage-endothelial cell immune signaling remains unexplored. In the current study we address this knowledge gap by characterizing cytokine secretion of macrophages, singularly and in co-culture with endothelial cells, during *T. pallidum* exposure. This study enhances understanding of the interactions between *T. pallidum*, macrophages, and endothelial cells, providing novel insight into host immune responses that contribute to *T. pallidum* dissemination, immune signaling, and symptoms of syphilis.

## 2 Methods

### 2.1 *Treponema pallidum* culture and sample preparation

*In vitro* cultures of *T. pallidum* with Sf1Ep cells were grown as previously described (Edmondson and Norris, 2021). To maintain the integrity of *T. pallidum* outer membrane proteins, treponemes were dissociated from Sf1Ep cells by incubation with trypsin-free dissociation media for 30 minutes at 34 °C in 1.5% O_2_, 5% CO_2_, and 93.5% N_2_ (Edmondson and Norris, 2021). To prepare Viable *Treponema pallidum* (VTP) samples (Waugh et al., 2023), Sf1Ep cells were removed by centrifugation at 220 x g, and *T. pallidum* in the supernatant were quantified by darkfield microscopy (Nikon Eclipse E600; Nikon Canada, Mississauga, Ontario) using a Petroff-Hauser counting chamber (Hauser Scientific, Horsham, PA) and diluted to achieve a multiplicity of infection (MOI) of 30 in 24-well tissue culture plates (Avantor, Radnor, PA). The infection extract control (IEC) was prepared by removing *T. pallidum* via syringe filtration of the diluted *T. pallidum* supernatant three times through a 0.22μm PES membrane (Avantor) (Waugh et al., 2023). Successful removal of *T. pallidum* via filtration was confirmed by darkfield microscopy, with no *T. pallidum* organisms observed in the filtered extract.

### 2.2 Mammalian cell culture

THP-1 cells (Cedarlane, Burlington, ON) were grown in RPMI-1640 medium (Cedarlane) supplemented with 10% fetal bovine serum (Corning) and 0.05mM 2-mercaptoethanol (henceforth referred to as ‘THP-1 complete medium’). THP-1 cells were differentiated to achieve a phenotype similar to monocyte-derived macrophages by incubation with 25ng/mL phorbol 12-myristate 13-acetate (PMA; Sigma-Aldrich, St. Louis, MO, USA) for 48 hours at 37 °C, 5% CO_2_, followed by a recovery period of 24 hours in fresh THP-1 complete media. Brain microvascular endothelial cells (hCMEC/d3, Cedarlane) were cultured as previously described (Waugh et al., 2023).

THP-1 cells were plated at a density of 2.4 x 10^5^ cells per well in 24-well tissue culture plates (Avantor) and differentiated into macrophages as described above. Macrophages were then exposed in triplicate to either VTP at a MOI of 30, IEC, or basal media (TpCM2, Edmondson and Norris, 2021) for 5, 12, 24, and 48 hours at 37°C, 1.5% O_2_, 5% CO_2_, 93.5% N_2_. At each timepoint, supernatant sample collection and *T. pallidum* viability confirmation were performed as previously detailed (Waugh et al., 2023). THP-1 and endothelial cell viability was assessed at 24 and 48 hours by trypan blue staining, with no significant difference in cell viability observed between treatment conditions (data not shown).

### 2.3 Macrophage and endothelial cell co-culture

hCMEC/d3 endothelial cells were passaged and resuspended in RPMI-1640 medium at a concentration of 2.4 x 10^5^ cells/mL. Similarly, macrophages were lifted and resuspended at a concentration of 2.4 x 10^5^ cells/mL in RPMI-1640 medium. The suspensions of THP-1 cells and HMBECs were then mixed to achieve a THP-1:HMBEC ratio of 1:1, plated in 24-well tissue culture plates at a density of 2.4 x 10^5^ cells/well, and incubated overnight at 37° in 5% CO_2_ to allow for adherence. Following overnight growth, the RPMI-1640 medium was removed, and cell cultures were exposed to VTP, IEC, or basal TpCM2, with supernatant samples collected at 5, 12, 24, and 48 hours post-exposure.

### 2.4 Cytokine analysis by cytometric bead arrays

Secretion of the cytokines IL-1β, IL-6, IL-8, IL-10, VEGF, Monocyte Chemoattractant Protein-1 (MCP-1), Regulated upon Activation, Normal T cell Expressed and Secreted (RANTES; CCL5), Granulocyte-Macrophage Colony-Stimulating Factor (GM-CSF), Tumor Necrosis Factor (TNF), and soluble Intracellular Adhesion Molecule 1 (sICAM-1) was quantified in supernatant samples using cytometric bead array flex sets (BD Biosciences, San Jose, California, USA) according to the manufacturer’s recommendations. For measurement of IL-6, RANTES, and TNF, culture supernatant was diluted 1:10 to ensure the measured cytokine concentrations fell within the standard curve for quantitation; for all other cytokines, neat culture supernatant was used for the assay. Raw data was acquired on a CytoFlex Flow Cytometer (Beckman Coulter, Mississauga, ON) and analyzed using CytExpert software (Beckman Coulter). Statistical analysis was performed using GraphPad Prism 9 by applying one-way ANOVA tests followed by Dunnett’s multiple comparisons to compare the mean of samples exposed to VTP to samples exposed to IEC and basal media. P-values ≤ 0.05 were considered statistically significant.

## 4 Results

### 4.1 *Treponema pallidum* exposure increased secretion of TNF, IL-1β, and IL-6 from macrophages and macrophage-endothelial co-cultures

We measured temporal cytokine secretion from macrophage-differentiated THP-1 cells exposed to *T. pallidum*, referred to here as “monoculture”, and macrophage-differentiated THP-1 cells in a 1:1 ratio with hCMEC/d3 human brain microvascular endothelial cells (HBMECs) exposed to *T. pallidum*, referred to here as “co-culture”. Cultures were exposed to either viable *T. pallidum* (VTP), infection extract control [IEC; to control for background *T. pallidum* culture components (Waugh et al., 2023)], or basal culture media.

**Figure 1:**
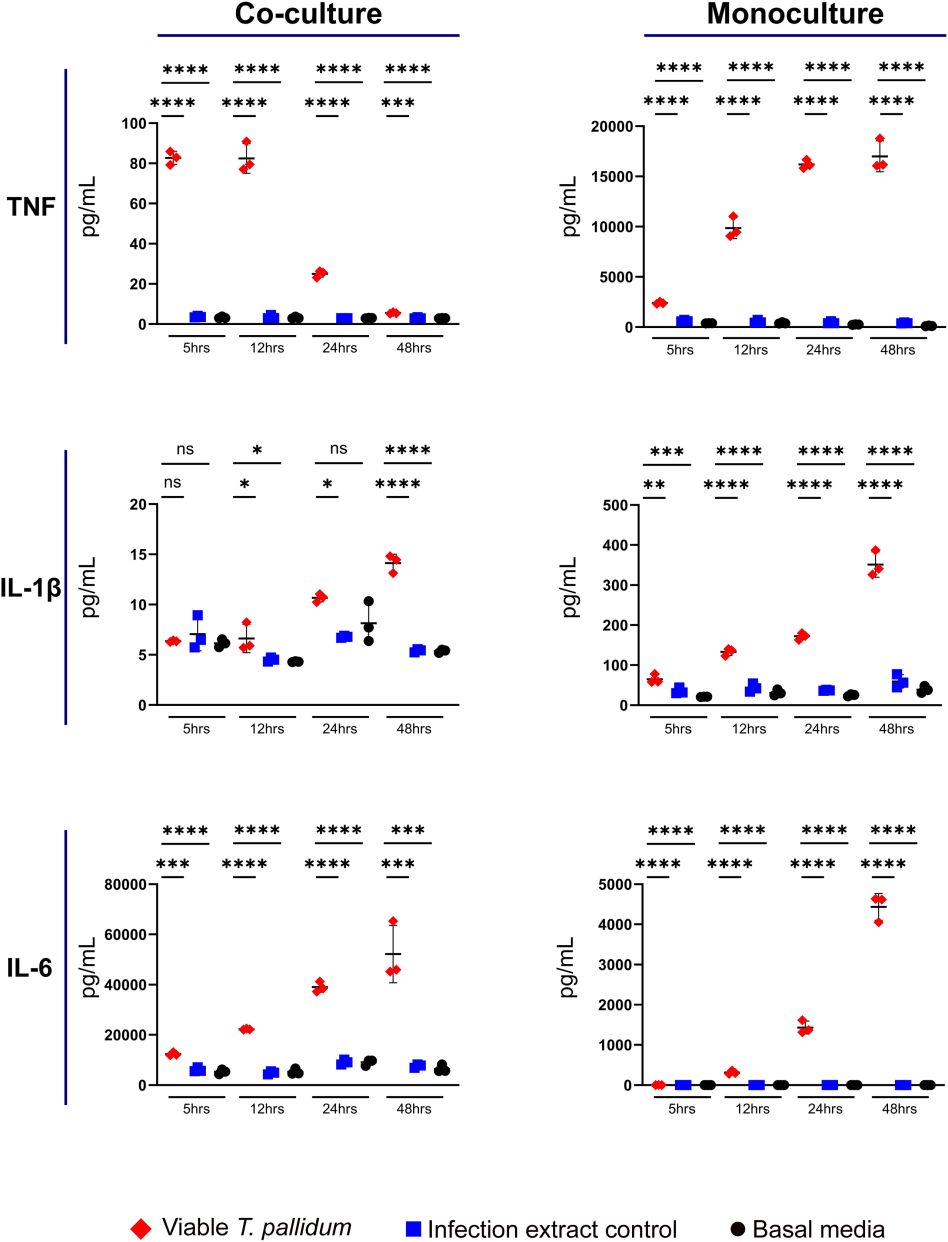
Supernatant concentrations of TNF, IL-1β, and IL-6 from macrophage-differentiated THP-1 cells in monoculture, or 1:1 co-culture with HMBECs, during exposure to *T. pallidum* (VTP) at an MOI of 30, infection extract control (IEC), or basal media for 5, 12, 24, or 48 hours. Data for each cytokine is representative of three experimental repeats; each replicate is shown in the **Supplementary Material**. Each timepoint represents a biological replicate, defined as an independent tissue culture well. The mean with standard deviation is shown. Statistical analysis was completed using a one-way ANOVA followed by Dunnetts multiple comparison. *p < 0.05, **p < 0.01, ***p < 0.001, ****p < 0.0001. Note the different Y-axis scales between co-cultures and monocultures.

Secretion of the pro-inflammatory cytokines TNF, IL-1β, and IL-6 were elevated in both the VTP-exposed monoculture and co-culture treatments, in comparison to the IEC and basal media control treatments (**Figure 1**, **Supplementary Figures S1**–**S3**). Additionally, TNF secretion increased over time in the VTP-exposed monocultures whereas the VTP-exposed co-cultures displayed decreased TNF secretion over time (**Figure 1**, **Supplementary Figure S1**). Secretion of IL-1β increased over time in both the VTP-exposed mono- and co-cultures (**Figure 1**, **Supplementary Figure S2**), as did secretion of IL-6 (**Figure 1**, **Supplementary Figure S3**). Finally, we observed minimal alteration in secretion of these cytokines in cultures exposed to the IEC and basal media controls, indicating that the secretion of these cytokines is specific to the presence of *T. pallidum*.

### 4.2 Secretion of VEGF, RANTES and soluble ICAM-1 is increased during *T. pallidum* exposure

**Figure 2:**
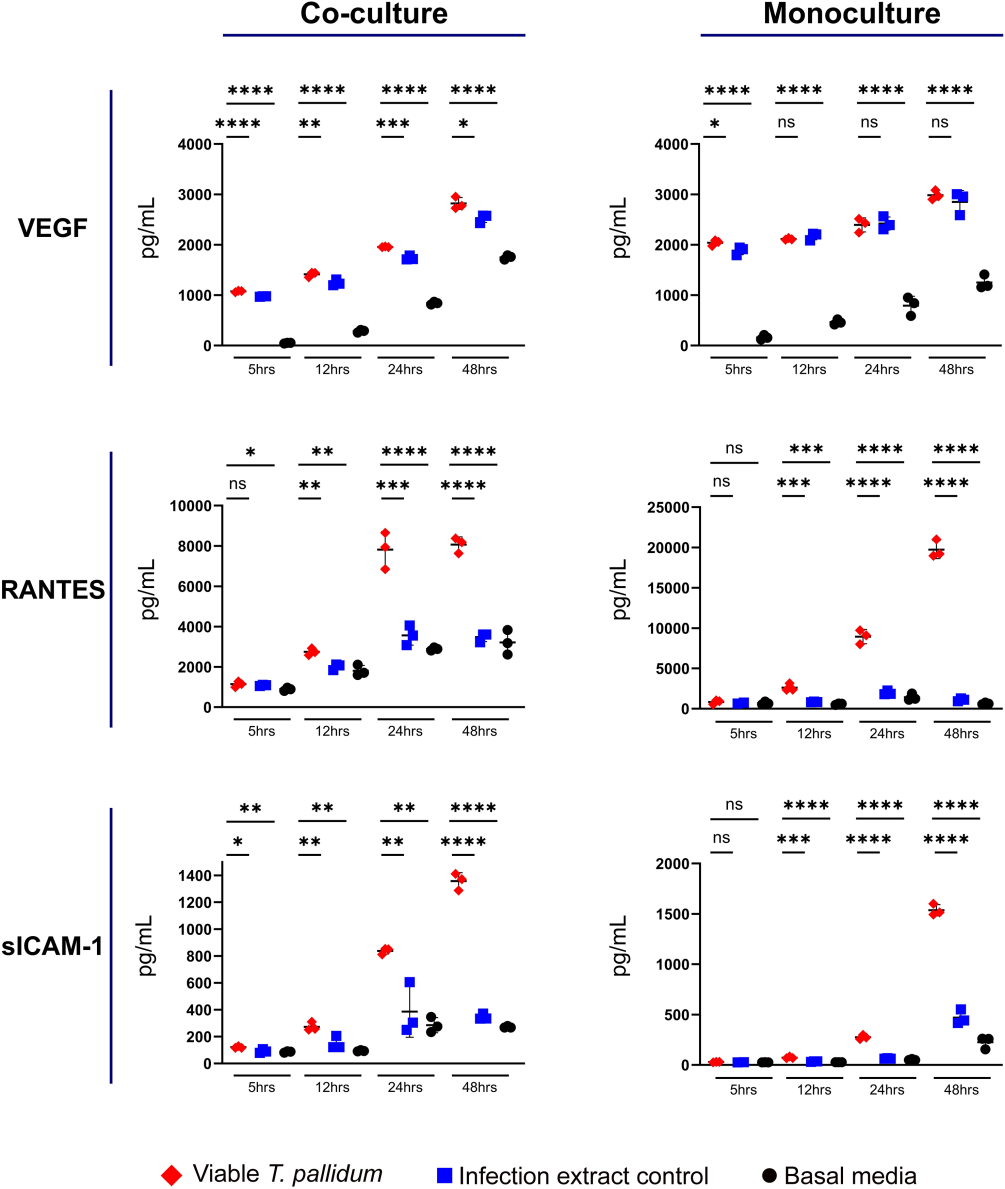
Supernatant concentrations of VEGF, RANTES, and sICAM-1 from macrophage-differentiated THP-1 cells in monoculture, or 1:1 co-culture with HMBECs, during exposure to *T. pallidum* (VTP) at an MOI of 30, infection extract control (IEC), or basal media for 5, 12, 24, or 48 hours. Data for each cytokine is representative of three experimental repeats; each replicate is shown in the **Supplementary Material**. Each timepoint represents a biological replicate, defined as an independent tissue culture well. The mean with standard deviation is shown. Statistical analysis was completed using a one-way ANOVA followed by Dunnetts multiple comparison. *p < 0.05, **p < 0.01, ***p < 0.001, ****p < 0.0001. Note the different Y-axis scales between co-cultures and monocultures.

We observed increased secretion of RANTES (CCL5) and sICAM-1 in both mono- and co-cultures exposed to VTP versus the control treatments, with secretion of VEGF modestly increased under co-culture conditions exposed to VTP (**Figure 2**; **Supplementary Figures S4**–**S6**). Analysis of these cytokines indicated variable levels of secretion in response to IEC-exposed cultures, indicating a non-*T. pallidum* specific response to components arising from the *T. pallidum* culture system. We observed RANTES secretion to be increased in VTP-exposed mono- and co-cultures (**Figure 2**; **Supplementary Figure S5**), with RANTES secretion increased over time under both culture conditions (**Figure 2**; **Supplementary Figure S5**). Secretion of sICAM-1 followed a similar trend, where mono- and co-cultures exposed to VTP secreted significantly greater sICAM-1 in comparison to control conditions, and where the secretion of sICAM-1 increased over time in VTP-exposed cultures (**Figure 2**; **Supplementary Figure S6**). Collectively, these data indicate that RANTES, and sICAM-1 are induced by *T. pallidum* during contact with macrophages and macrophage-endothelial co-cultures. Similar to our previous studies (Waugh et al., 2023), these data indicate the importance of including proper controls when studying *T. pallidum*-host interactions.

### 4.3 Secretion of monocyte recruiting and activating cytokines is increased during *T. pallidum* exposure

**Figure 3:**
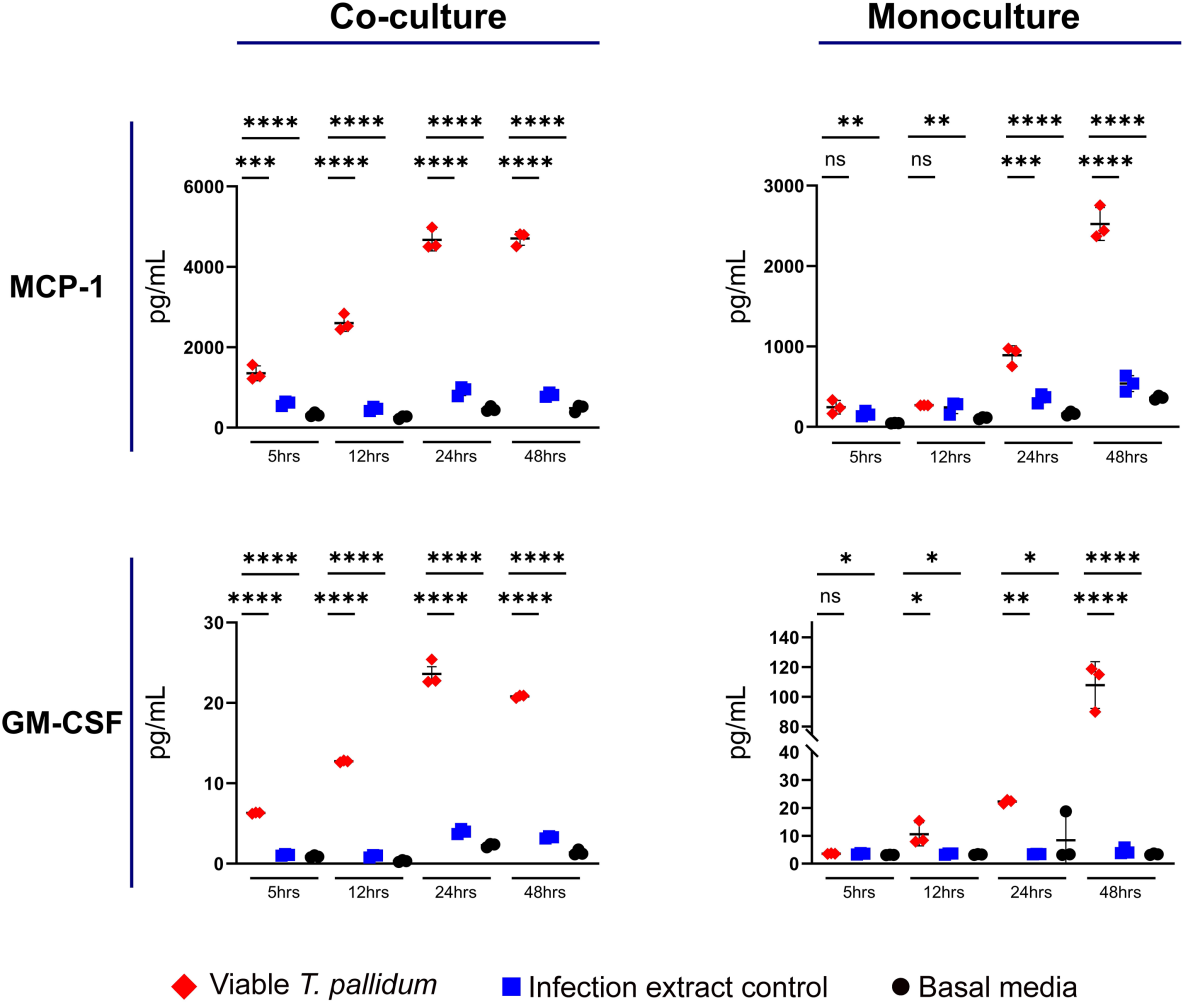
Supernatant concentrations of MCP-1 and GM-CSF from macrophage-differentiated THP-1 cells in monoculture, or 1:1 co-culture with HMBECs, during exposure to *T. pallidum* (VTP) at an MOI of 30, infection extract control (IEC), or basal media for 5, 12, 24, or 48 hours. Data for each cytokine is representative of three experimental repeats; each replicate is shown in the **Supplementary Material**. Each timepoint represents a biological replicate, defined as an independent tissue culture well. The mean with standard deviation is shown. Statistical analysis was completed using a one-way ANOVA followed by Dunnetts multiple comparison. *p < 0.05, **p < 0.01, ***p < 0.001, ****p < 0.0001. Note the different Y-axis scales between co-cultures and monocultures.

We observed that VTP exposure increased secretion of MCP-1 and GM-CSF in both the mono- and co-cultures (**Figure 3**; **Supplementary Figures S7**, **S8**). In the monocultures, significant induction of MCP-1 during VTP exposure over controls was not observed until the 24-hour timepoint, indicating the delayed induction of this cytokine by macrophages alone. In the co-cultures, MCP-1 secretion was significantly increased in VTP*-*exposed samples versus the controls by the 5 and 12 hour timepoints (**Figure 3**; **Supplementary Figure S7**). Secretion of GM-CSF demonstrated similar trends, where VTP-exposed cultures increased secretion of GM-CSF over time (**Figure 3**; **Supplementary Figure S8**). These data demonstrate that *T. pallidum* induces MCP-1 and GM-CSF secretion in both macrophage monocultures and macrophage-endothelial co-cultures, an induction that increases over time in both culture conditions.

### 4.4 IL-8 secretion is decreased in macrophages and macrophage-endothelial co-cultures exposed to *T. pallidum*

**Figure 4:**
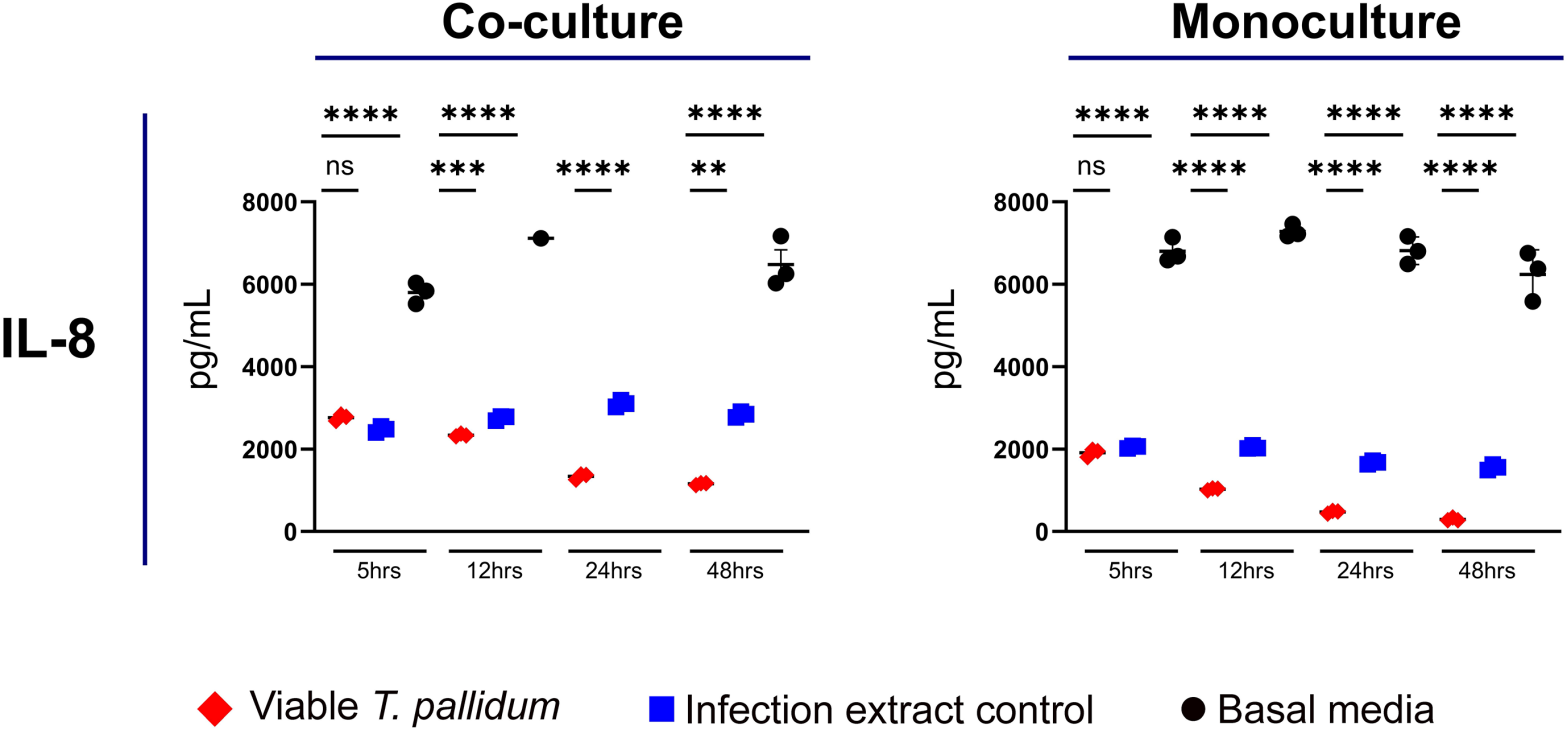
Supernatant concentrations of IL-8 from macrophage-differentiated THP-1 cells in monoculture, or 1:1 co-culture with HMBECs, during exposure to *T. pallidum* (VTP) at a MOI of 30, infection extract control (IEC), or basal media for 5, 12, 24, or 48 hours. Data for each cytokine in representative of three experimental repeats; each replicate is shown in the **Supplementary Material**. Each timepoint represents a biological replicate, defined as an independent tissue culture well. The mean with standard deviation is shown. For the basal media-exposed co-cultures, at the 12 hour timepoint two replicates were above the upper limit of quantitation, and at 24 hours all replicates were above the upper limit of quantitation, therefore these datapoints are not shown in the Figure. Statistical analysis was completed using a one-way ANOVA followed by Dunnetts multiple comparison. **p < 0.01, ***p < 0.001, ****p < 0.0001.

We observed that VTP-exposed mono- and co-cultures had reduced secretion of IL-8 relative to both the basal media and IEC control exposures (**Figure 4**; **Supplementary Figure S9**). In both the mono- and co-culture systems, the basal media treatment resulted in the greatest secretion of IL-8 (**Figure 4**; **Supplementary Figure S9**). These data show that both *T. pallidum* and components found in the *T. pallidum* culture system decrease IL-8 secretion in our culture systems, with the presence of viable *T. pallidum* reducing IL-8 secretion to the greatest extent.

## 5 Discussion

Endothelial cells and macrophages are important cell types that *T. pallidum* encounters during all stages of infection. Endothelial cells assist in directing innate immune responses, including through recruiting and enabling extravasation of leukocytes (Amersfoort et al., 2022). Further, endothelial cells form the vascular barrier through which *T. pallidum* traverses during bacterial dissemination to distant locales in the body. Macrophages are an important contributor to clearance of *T. pallidum* from sites of infection, and both endothelial cells and macrophages undergo bidirectional signaling to influence function and activity in the other cell type. Endothelial-macrophage interactions are involved in regulating vascular homeostasis, angiogenesis, innate immunity, and in bridging adaptive and innate immune responses (Baer et al., 2013; Amersfoort et al., 2022). Despite this diverse array of interactions and functional relevance to *T. pallidum* infection, understanding of how these two infection-relevant cell types respond on a molecular level to *T. pallidum* exposure is incomplete. Here we begin to address this knowledge gap by profiling the secretion of a panel of ten cytokines from macrophage-differentiated THP-1 cells in monoculture, and in 1:1 co-culture with human brain microvascular endothelial cells during exposure to viable *T. pallidum*.

In the current study we observed increased secretion of the pro-inflammatory cytokines TNF, IL-1β, and IL-6 in mono- and co-culture conditions in response to *T. pallidum*. Previous investigations reported increased TNF and IL-6 secretion in human macrophages (Hawley et al., 2017) and HBMECS (Waugh et al., 2023) during *T. pallidum* exposure, findings which are supported by this study, thus identifying these cytokines as important regulators of both the endothelial and macrophage innate immune response to *T. pallidum.* At endothelial sites, IL-6, TNF, and IL-1β downregulate tight junction proteins and promotes junctional relaxation, which may enhance *T. pallidum* dissemination (Paul et al., 2003; O’Carroll et al., 2015; Blecharz-Lang et al., 2018). Previous studies investigating macrophage cytokine secretion did not report increased IL-1β secretion (Hawley et al., 2017), whereas in the current study IL-1β secretion was increased in *T. pallidum*-exposed macrophage mono- and co-cultures. The variation in these findings may result from different macrophage sources (Primary vs. THP-1), methods of macrophage activation (IFN-γ vs. PMA), or sampling collection times (8 hours vs. 5–48 hours) (Hawley et al., 2017). Also of interest, in the current study a temporal trend of decreased TNF secretion was observed in co-culture conditions, while increasing TNF secretion was observed over time in monoculture conditions. The reason for this divergent result is not currently understood, but may suggest that endothelial cells have a moderating effect on TNF secretion from macrophages when exposed to *T. pallidum*.

We also observed increased secretion of VEGF, RANTES, and sICAM-1 in VTP-exposed mono- and co-cultures, relative to the controls, with concentrations increasing over time. Previous studies identified increased secretion and expression of VEGF in HMBECs exposed to *T. pallidum* (Waugh et al., 2023; Waugh et al., 2025), and VEGF is increased in secondary syphilis lesions (Macaron et al., 2003). However, our study revealed that IEC and basal media exposures also induced significant and variable secretion of these cytokines. These data suggest that secreted *T. pallidum* components and/or materials resulting from Sf1Ep cell culture influence secretion of these cytokines. Further, prior studies have shown that *T. pallidum*-exposed macrophages release exosomes that act upon endothelial cells to promote secretion of ICAM-1, Vascular Cell Adhesion Protein 1 (VCAM-1), VEGF, and IL-8 (Xu et al., 2019). Increased levels of VEGF have been observed in angiogenic cutaneous syphilis lesions, suggesting a role for VEGF in remodeling endothelial and tissue sites during infection (Macaron et al., 2003).

Our observation in the current study that macrophages secrete RANTES in response to *T. pallidum* exposure is of interest considering the involvement of RANTES in recruitment of leukocytes to the vasculature and arrest of monocytes and macrophages at endothelial sites (Baltus et al., 2003). Importantly, RANTES increases blood-brain barrier permeability and endothelial dysfunction (Terao et al., 2008). Therefore, by inference, the high levels of RANTES secretion in VTP-exposed mono- and co-cultures observed in the current study is expected to contribute to endothelial barrier dysfunction. This inflammatory milieu may also enhance *T. pallidum* traversal across endothelial barriers, including the blood-brain barrier. Previous reports show that *T. pallidum* and *T. pallidum*-derived proteins induce expression of the RANTES receptor, C-C chemokine receptor type 5 (CCR5), in peripheral blood mononuclear cells (Sellati et al., 2000). Interestingly, increased expression of both RANTES and CCR5 facilitate the pathogenesis of Human Immunodeficiency Virus (HIV) due to the role of CCR5 as a HIV co-receptor on both macrophages and T cells (Gornalusse et al., 2015). *Treponema pallidum*-induced secretion of RANTES may facilitate the development of a favorable endothelial microenvironment for HIV to persist and infect both T cells and macrophages in individuals infected with *T. pallidum*, offering a potential explanation for the frequent incidence of syphilis-HIV co-infections.

sICAM-1 is an inflammatory biomarker expressed as a spliced isoform of membrane-bound ICAM-1 and created as a product of proteolytic cleavage of ICAM-1 (King et al., 1995; Bui et al., 2020). sICAM-1 is released by activated endothelial cells and macrophages, particularly in response to IL-6 and TNF stimulation (Bui et al., 2020). Increased secretion of sICAM-1 is implicated in multiple inflammatory diseases of infectious and non-infectious origin (Bui et al., 2020), including sepsis (Hedetoft et al., 2021). Further, increased sICAM-1 secretion has been shown to promote angiogenesis, enhance endothelial motility, and inhibit leukocyte-endothelial interactions (Rieckmann et al., 1995), with increased serum concentrations of sICAM-1 documented in blood-cerebrospinal barrier dysfunction and acute bacterial meningitis (Rieckmann et al., 1993). Thus, the consequence of increased sICAM-1 in the context of a syphilis infection may include attenuating endothelial-macrophage interactions and contributing to an inflammatory response that disrupts endothelial barrier integrity. Other neuroinvasive bacteria, including *Streptococcus suis*, have been shown to induce secretion of sICAM-1 (Grenier and Bodet, 2008), suggesting possible convergent mechanisms of endothelial barrier disruption between *T. pallidum* and other neuroinvasive pathogens.

Induction of secretion of MCP-1 and GM-CSF was observed in both VTP-exposed mono- and co-cultures, a situation which would assist with recruiting and activating monocytes at sites of infection. Conversely, previously we have shown reduced MCP-1 secretion in IL-32γ-activated macrophages exposed to *T. pallidum*-derived proteins (Houston et al., 2022) and brain endothelial cells exposed to *T. pallidum* (Waugh et al., 2023). These divergent results highlight the importance of environmental context, with immune responses to *T. pallidum* anticipated to be influenced by the presence of co-localized immune cells and direct pathogen-host cell engagement.

In this study mono- and co-cultures exposed to *T. pallidum* had significantly reduced IL-8 secretion compared to controls, while our previous investigations found increased IL-8 secretion by *T. pallidum*-exposed brain endothelial cells cultured in the absence of macrophages (Waugh et al., 2023). Similarly, IL-8 secretion was observed to increase in our previous investigation of IL-32γ-activated macrophages exposed to *T. pallidum* proteins (Houston et al., 2022), as well as a prior study conducted by Pozzobon et al. investigating human umbilical vein endothelial cells exposed to a recombinant *T. pallidum* protein (Pozzobon et al., 2016). These data suggest that the extent of expression of this cytokine can be influenced by multiple factors, including direct *T. pallidum* engagement rather than the use of recombinant *T. pallidum* proteins, the methodology used for macrophage activation, and the presence of additional host cell types.

Here we report time course secretion profiles of THP-1 macrophages alone and in 1:1 co-culture with HMBECs during exposure to *T. pallidum*. To our knowledge, this is the first study to explore *in vitro* macrophage-endothelial co-cultures in the presence of *T. pallidum*. While our study expands knowledge on host responses to *T. pallidum* beyond conventional single host cell conditions, it has limitations. These include the use of PMA to activate THP-1 cells, which results in differentiation of macrophages into an M0 lineage (Tedesco et al., 2018). This macrophage phenotype may exhibit non-biologically relevant effects, exemplified by the observed high baseline secretion of IL-8 observed in the current study and previously reported in PMA-treated THP-1 cells (Kämpfer et al., 2017). An additional limitation is that equal numbers of endothelial cells and macrophages were seeded in the co-culture conditions, thus resulting in a higher number of macrophages seeded in the monoculture conditions. These experimental conditions were required to ensure sufficient cell numbers in the plate wells, and consistent total cell numbers between conditions. Therefore, in this study we did not make direct comparisons of cytokine secretions between monocultures and co-cultures. Further, our *in vitro* model will not provide a comprehensive readout of the immune response to *T. pallidum*, since during an *in vivo* infection additional cell types and inflammatory mediators are present. Also, in our experimental design we are unable to exclude secreted *T. pallidum* components and products from lysed treponemes in our infection extract control, which may affect the interpretation of our results. However, despite these limitations the studies presented here expand on previous findings detailing increased secretion of IL-6 and TNF (Hawley et al., 2017) and report the secretion of RANTES, sICAM-1, IL-1β, GM-CSF, and MCP-1 in macrophages and macrophage-endothelial co-cultures during *T. pallidum* exposure. These findings further our understanding of temporal cytokine responses to *T. pallidum* and identify immune signaling factors induced during the host immune response to *T. pallidum* infection.

## Data Availability

The original contributions presented in the study are included in the article/**Supplementary Material**. Further inquiries can be directed to the corresponding author.
