# Supplementary material for: Treponema pallidum induces pro-inflammatory cytokine secretion in macrophages and macrophage-endothelial co-cultures
Source: Front Cell Infect Microbiol. 2025 Oct 17;15:1681813. doi: 10.3389/fcimb.2025.1681813 (PMC12575358; doi:10.3389/fcimb.2025.1681813)
Supplement: Supplementary file 4 [file DataSheet4.pdf]

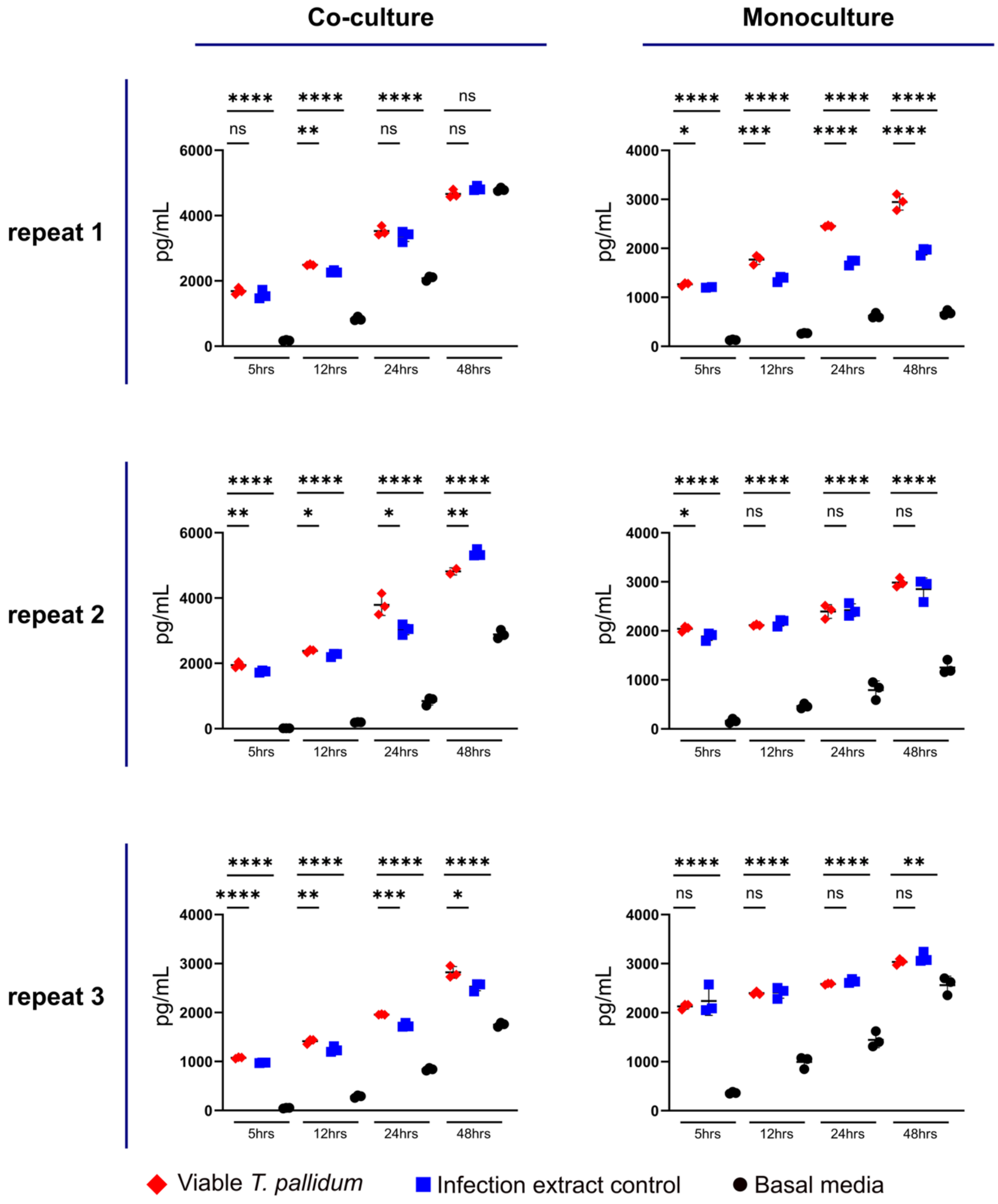

Supplementary Figure 4. Supernatant concentrations of VEGF from macrophage-differentiated THP-1 cells in monoculture, or 1:1 co-culture with HMBECs, during exposure to *T. pallidum* (VTP) at a MOI of 30, infection extract control (IEC), or basal media for 5, 12, 24, or 48 hours. Data for each cytokine is representative of three experimental repeats, and a representative replicate is shown in Figure 2. Each timepoint represents a biological replicate, defined as an independent tissue culture well. The mean with standard deviation is shown. Statistical analysis was completed using a one-way ANOVA followed by Dunnetts multiple comparison. * $p < 0.05$, **$p < 0.01$, ***$p < 0.001$, ****$p < 0.0001$.
