# Supplementary material for: Treponema pallidum induces pro-inflammatory cytokine secretion in macrophages and macrophage-endothelial co-cultures
Source: Front Cell Infect Microbiol. 2025 Oct 17;15:1681813. doi: 10.3389/fcimb.2025.1681813 (PMC12575358; doi:10.3389/fcimb.2025.1681813)
Supplement: Supplementary file 9 [file DataSheet9.pdf]

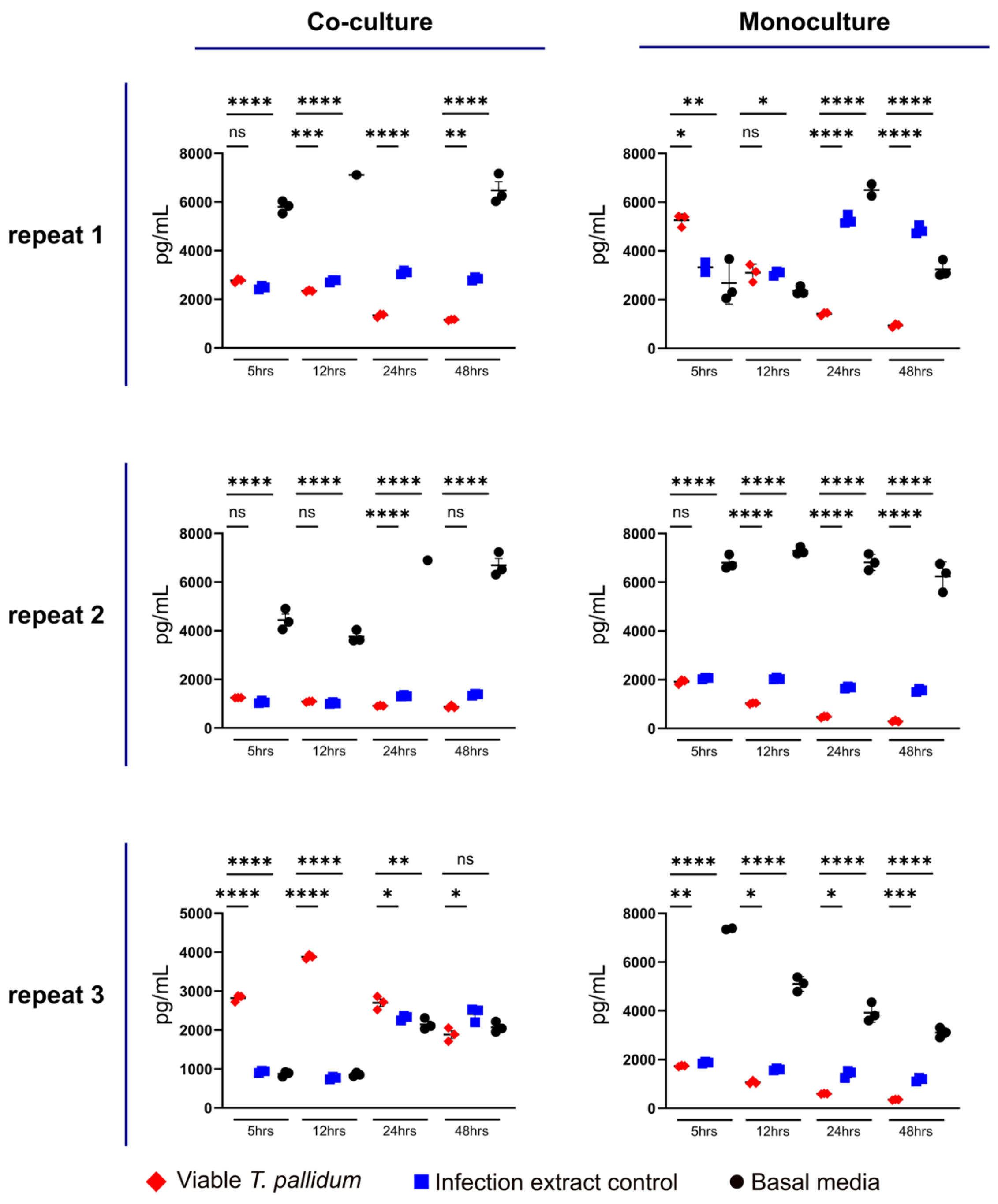

Supplementary Figure 9. Supernatant concentrations of IL-8 from macrophage-differentiated THP-1 cells in monoculture, or 1:1 co-culture with HMBECs, during exposure to *T. pallidum* (VTP) at a MOI of 30, infection extract control (IEC), or basal media for 5, 12, 24, or 48 hours. Data for each cytokine is representative of three experimental repeats, and a representative replicate is shown in Figure 4. Each timepoint represents a biological replicate, defined as an independent tissue culture well. For the basal media-exposed co-cultures in repeat one, at the 12 hour timepoint two replicates were above the upper limit of quantitation, and at 24 hours all replicates were above the upper limit of quantitation. Similarly, in repeat two at the 24 hour timepoint, two replicates of the basal media-exposed co-cultures were above the limit of quantitation. Therefore, these datapoints are not shown in the figure.The mean with standard deviation is shown. Statistical analysis was completed using a one-way ANOVA followed by Dunnetts multiple comparison. * $p < 0.05$, ** $p < 0.01$, *** $p < 0.001$, **** $p < 0.0001$.
